# Editorial: Advancements and challenges in mental health services: 2022

**DOI:** 10.3389/frhs.2023.1348169

**Published:** 2023-12-21

**Authors:** Carolyn S. Dewa

**Affiliations:** Department of Psychiatry and Behavioral Sciences, University of California, Davis, Sacramento, CA, United States

**Keywords:** mental health services, acceptability of care, supply and distribution of providers, availability of services, non-financial costs, system barriers to access

**Editorial on the Research Topic** Advancements and challenges in mental health services: 2022

Significant unmet mental health service needs exist across the globe (1). Barriers contributing to these gaps in care include finances, mental health provider supply and distribution, and stigma (1). Seeking to address unmet needs, our *Advancements and Challenges in Mental Health* research topic aims to call attention to studies on (1) new and novel mental health service and support models that extend access and treatment and (2) current gaps and barriers to mental health services and supports. In 2022, six papers were published on this topic. These publications reflect the breadth of the populations served by mental health service programs. Together, they also offer insights into common challenges novel mental health services face. One major impediment is translating availability into access. This paper collection elucidates some of the barriers preventing straightforward translation; they include (1) acceptability to service users, (2) acceptability to providers, (3) system barriers, and (4) non-financial costs.

## Availability and access

In this collection, two studies examine the effects of extending mental health services accessibility through two different mechanisms. Palay et al. study focuses on outcomes of a novel post-crisis program for people who use emergency/crisis services and do not require an inpatient admission but have high acuity. Offering substitute specialist group visits, the new program facilitated access to post-crisis services bypassing the usual 2–4-week wait for a solo specialist appointment. The results were promising. However, over 14 months, 48 people enrolled in the new program. This suggests that availability alone does not ensure wide use of a program. These results raise questions about the characteristics of those who would attend group visits in lieu of individual visits and factors that attract people to rely on group visits.

Yoon et al. examine the effects of expanded health insurance coverage on pregnant and post-partum women's access to depression screening and treatment for those enrolled in Oregon's Medicaid program. In the US, lack of health insurance coverage can make services unaffordable and inaccessible. They found that expanded insurance benefits were associated with significant increases in overall depression screening and treatment. However, access differed by race/ethnicity and geographic location. These results are reminders that finances alone do not determine access for everyone. They suggest the existence of additional barriers to depression screening and treatment confront women from various racial/ethnic groups who live in areas with limited healthcare provider access.

## Acceptability to service users

Cheng et al.'s study highlights barriers and needs related to early access and treatment of psychosis identified by youth, family caregivers, and service providers. Some barriers result from treatment refusal because youths do not recognize they need it. Among members of Indigenous communities, additional barriers result from mistrust of the mental health system due to historical injustices and discrimination. These results reinforce the importance of WHO's *Guidelines on Mental Health Promotive and Preventive Interventions for Adolescents* (2), emphasizing access to culturally appropriate services.

## Acceptability to service providers

Service providers play important roles in access. Analyzing audio work sample recordings, Sibley et al. examine community provider provision of evidence-based non-pharmacologic treatments to adolescent clients with Attention-Deficit/Hyperactivity Disorder. Significant gaps involved evidence-based practices requiring parent participation. These results raise questions about how clinicians choose their practices and the role of clients and their parents in the decision. Although the quantitative results did not reveal any predictors of evidence-based practice implementation, it is not clear whether the study was sufficiently powered for the logistic regression model and the number of independent variables included. Future studies could increase the sample size and improve the generalizability of the results by broadening geographic scope.

Forchuk et al. conducted qualitative interviews with healthcare and service providers to investigate barriers to incorporating methamphetamine use harm reduction (MUHR) into hospital practices. Identified barriers related to provider acceptance of these strategies and mistrust between clinicians and service users. As with Cheng et al., Forchuk et al. identified trust as critical to access.

## System barriers

System structure barriers were also highlighted. Palay et al. point out that system characteristics influence access to new and novel services. For example, systems designed to reimburse providers for individual visits will not have mechanisms to reimburse substitute group visits. To further inform funding decisions, outcomes that directly impact funding such as comparisons of emergency department/crisis services recidivism rates for the novel intervention compared with usual care could be examined.

Forchuk et al. found that system barriers to the adoption of MUHR strategies included substance unavailability to providers. Increased costs of space, specialized staff to support these strategies, and managing associated risks are also barriers. In another study, Forchuk et al. found that to manage risks, hospitals institute policies hindering MUHR adoption. For instance, hospitals require sharp box removal from rooms of patients who use illicit substances to enforce illicit drug abstinence. However, these policies also expose staff to the dangers of sticks from illicitly used syringes.

## Non-financial costs

Non-financial costs related to insufficient provider supply and distribution also create barriers. Cheng et al. found study participants living in remote and rural areas described barriers related to insufficient supply and distribution of mental health providers can result in a mental health system that is isolated and disconnected. It forces service users and their caregivers to travel great distances to access care. Program scarcity also makes it difficult to create a system that supports people to avoid a crisis point rather than exposing them and their families to the trauma of a crisis. Provider scarcity can add to the difficulty of accessing evidence-based care such as the Early Psychosis Intervention program model that calls for a multi-disciplinary team approach.

Palay et al. also discuss challenges related to provider scarcity. While specialist group visits could offer post-crisis clients quicker access to specialists, there was a shortage of supplemental community programs to support clients.

## Solutions

Cheng et al. and Forchuk et al. searched for ways to translate knowledge into access. Both stress the importance of stakeholder education. They also underscore the need for cross-sector collaboration as well as provider and system acceptance of novel programs and effective practice recommendations. Cheng et al. suggest the use of service mandates to emphasize the specific needs of populations with high rates of unmet needs.

## Conclusions

This collection offers hope of effective new programs and solutions that extend access to services that address the complex unmet needs of people experiencing mental illnesses. They also suggest future studies, shining light on the importance of including outcomes that may be of interest to decision-makers and stakeholders in the design of an experimental program and study.
